# A multi-perspective qualitative exploration of the reasons for changes in the physical activity among 10–11-year-old children following the easing of the COVID-19 lockdown in the UK in 2021

**DOI:** 10.1186/s12966-022-01356-3

**Published:** 2022-09-05

**Authors:** Robert Walker, Danielle House, Lydia Emm-Collison, Ruth Salway, Byron Tibbitts, Kate Sansum, Tom Reid, Katie Breheny, Sarah Churchward, Joanna G. Williams, Frank de Vocht, William Hollingworth, Charlie Foster, Russell Jago

**Affiliations:** 1 Centre for Exercise, Nutrition & Health Sciences, School for Policy Studies, University of Bristol, Bristol, BS8 ITZ UK; 2 Population Health Sciences, Bristol Medical School, University of Bristol, Bristol, BS8 2PS UK; 3 Independent Public Member of the Project Team, Bristol, UK; 4 Communities and Public Health, Bristol City Council, Bristol, BS1 9NE UK; 5 The National Institute for Health Research, Applied Research Collaboration West (NIHR ARC West), University Hospitals Bristol and Weston NHS Foundation Trust, Bristol, BS1 2NT UK; 6 NIHR Bristol Biomedical Research Centre, University Hospitals Bristol and Weston NHS Foundation Trust and University of Bristol, Bristol, UK

**Keywords:** Coronavirus, Pandemic, Health, Child, Exercise, Sport, Interviews, Focus groups, Framework method

## Abstract

**Background:**

Active-6 is exploring how the COVID-19 pandemic has impacted physical activity behaviour among Year 6 children (aged 10–11 years) and their parents in Southwest England. Initial findings from the Active-6 project have shown a 7–8 min decrease in moderate-to-vigorous physical activity and an increase in sedentary behaviour among children following the easing of restrictions in the UK in latter half of 2021. This finding suggests that the pandemic has had a persistent impact on child physical activity behaviour. This paper explored the possible mechanisms behind these changes.

**Methods:**

Interviews with parents (*n* = 21), members of school staff (*n* = 9) and focus groups with children aged 10–11 years (*n* = 47) were conducted between August and December 2021 to discuss the impact of the pandemic on child physical activity behaviour. The framework method was used for analysis.

**Results:**

Five themes spanning two key stages of the pandemic were described. Three themes related to the period of lockdowns and fluctuating restrictions (March 2020 – April 2021). These included: Theme 1) Lockdown: A short-lived adventure; Theme 2) Access to facilities during restrictions; and Theme 3) The importance of the parent. A further two themes were identified related to the period following the gradual easing of restrictions in April 2021. These included: Theme 4) An overwhelming return to normal; and Theme 5) Reopening fatigue.

**Conclusions:**

The analysis suggested that feelings of novelty experienced during the initial stages of lockdown waned as restrictions were prolonged, creating an increasingly challenging period for parents and their children. However, during periods of restrictions, the importance of parental encouragement and access to appropriate facilities in the local and home environment helped facilitate physical activity. Following the easing of COVID-19 restrictions, emotional overwhelm and physical fatigue among children, stemming from a sedentary and socially isolated life in lockdown and other restrictions, were key contributors to the decreased moderate to vigorous physical activity and increased sedentary behaviour that was observed in a related quantitative study.

**Supplementary Information:**

The online version contains supplementary material available at 10.1186/s12966-022-01356-3.

## Background

Physical activity has been associated with a number of physical and mental health benefits among children, including decreased risk of depression, cardiometabolic risk factors, and increased executive functioning, attention, and academic performance [1–4]. Studies have shown that large proportions of children and young people do not meet the public health guidance related to physical activity [5–8]. The World Health Organisation and UK Chief Medical Officers recommend that children and young people should engage in an average of an hour of moderate-to-vigorous intensity physical activity (MVPA) per day, accumulated across the day [9–11]. However, prior to the pandemic, it was estimated that only 41% of children aged 10–11 years in the UK met this recommendation [5]. The COVID-19 pandemic has impacted physical activity behaviour among many populations [12, 13], including children in the UK [14, 15], USA [16], Italy [17], and the Netherlands [18], and its long-term effects are unknown.

**Fig. 1:**
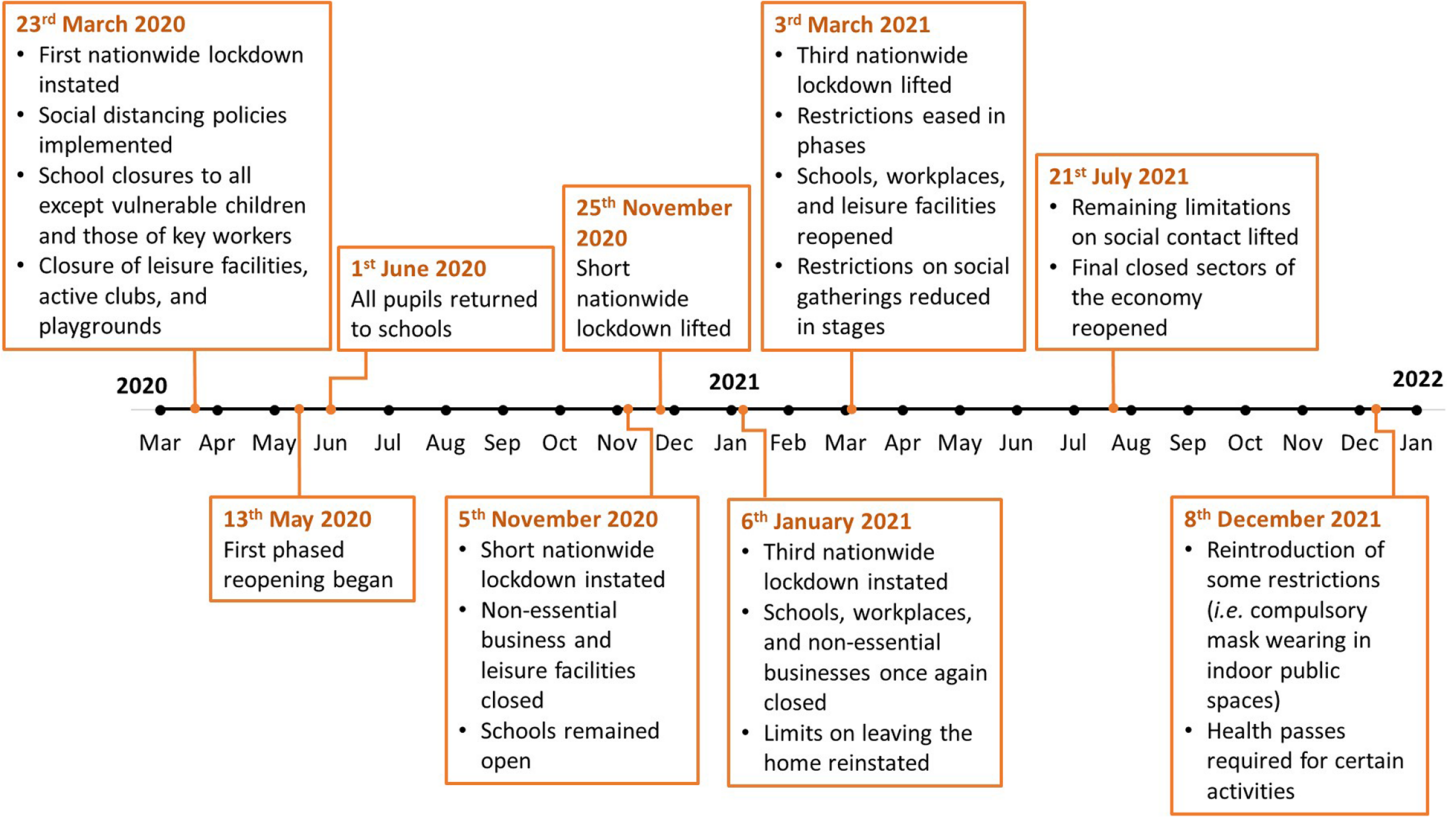
Timeline of UK Government coronavirus lockdowns and measures (March 2020 to December 2021)

People living in England between March 2020 and December 2021 experienced varying levels of rules and restrictions aimed at reducing the spread of COVID-19 [19]. Figure 1 illustrates the timeline and key periods of government coronavirus lockdowns and restrictions in England. The Active-6 project [20] seeks to examine the impact of changes that have resulted from the COVID-19 pandemic on physical activity behaviour among Year 6 children (aged 10–11 years) and their parents/guardians. Using accelerometer data collected between May and December 2021 we found that Year 6 children were taking part in an average of 7–8 fewer minutes of MVPA and 25 additional minutes of sedentary time per day than a pre-pandemic comparator group who were recruited from the same schools and assessed using the same methods before the pandemic [14]. These results indicate that the detrimental impact of the pandemic on children’s physical activity appears to continue beyond the immediate period of actual restrictions. This is particularly concerning as children’s MVPA has been shown to decline at a rate of 2.2 min per year [5], suggesting the expected decline in MVPA has accelerated by three years. Evidence surrounding longer-term trajectories is limited, and how this accelerated decline in MVPA will impact future physical activity behaviour is yet unknown. The importance of physical activity participation for health and wellbeing over the life-course are well established [10, 11], with reductions caused by the COVID-19 pandemic having the potential to cause significant future public health issues. Thus, to inform future strategies to address reductions in physical activity, it is important to understand what factors may have contributed to its decline. The aim of this study is to explore reasons behind the reduction in child physical activity observed in a related study using the perspectives of parents/guardians, children, and primary school staff.

## Methods

### Participants and procedure

Data were collected from three groups participating in the Active-6 project: 1) Six focus groups were conducted with 47 children from six schools; 2) one-to-one semi-structured interviews were conducted with 21 parents who were recruited from 13 schools; and 3) one-to-one semi-structured interviews were conducted with nine members of school staff recruited from nine schools. Parents and children who had worn accelerometers as part of the Active-6 project and had consented to being recontacted were invited to participate in an interview or focus group. Staff from schools who were involved in the Active-6 project were directly contacted and invited to participate. Children and parents were individually categorised as low, medium, or high MVPA level based on their accelerometer-measured weekday MVPA in relation to other participants in their school group. Even ratios of low/medium/high MVPA children were invited from an even range of schools situated in urban/rural and high/low deprivation areas. Index of Multiple Deprivation (IMD; http://data.gov.uk/dataset/index-of-multiple-deprivation) was determined from postcode of the school or parent provided during the study sign-up process. Higher IMD score indicates a greater level of deprivation and are displayed as deciles. Participant demographic information can be seen in Table 1.

**Table 1:** Characteristics of Active -6 interview and focus group participants by demographic, physical activity and job role

	N
**Parents**	**21**
Gender	
Male	0
Female	21
Parent activity levels	
High MVPA	11
Medium MVPA	9
Low MVPA	1
Age (years)	
30–34	1
35–39	2
40–44	11
45–49	7
IMD decile	
≤ 5	4
> 5	17
**School staff**	**9**
Gender	
Male	3
Female	6
Role	
Year 6 teacher	7
Full-time PE Coordinator	1
Deputy Headteacher	1
**Children**	**47**
Gender	
Male	26
Female	21
Child activity levels	
High MVPA	16
Medium MVPA	16
Low MVPA	15

*Note. IMD decile* ≤ 5 = greater level of deprivation, > 5 = lesser level of deprivation

Parents were interviewed between September and December 2021, school staff between November and December 2021, and focus groups were conducted in December 2021. All interviews were conducted remotely by RW, TR, and BT, while focus groups were conducted in person at the children’s schools by RW, TR, BT, and DH. Parent interviews ranged from 27 to 75 min in duration, school contact interviews from 33 to 59 min, and child focus groups from 33 to 61 min. Parents and school staff were provided a £10 gift voucher as recompense for their time. Ethical approval was gained from the School of Policy Studies Ethics Committee at the University of Bristol, UK (Ref SPSREC/20–21/150). Written informed consent was obtained for all participants with children providing assent [21].

### Study materials

Separate interview/focus group topic guides were developed for each participant group. Topic guides focused upon changes to physical activity behaviour among parents and children over four key time periods of the COVID-19 pandemic: 1) the first school closure (March—June 2020); 2) the first reopening of schools (June 2020—January 2021); 3) the second school closure (January – April 2021); and 4) the second reopening of schools (post-April 2021). Parents were asked how their own, and their child’s activity patterns had changed and factors that had influenced any changes. School contact interviews focused upon changes within the school environment that might have influenced activity levels among Year 6 pupils. The research team anticipated challenges with temporal memory among children. Subsequently, rather than focusing on specific timepoints over the course of the pandemic, the focus group topic guide was centred around pandemic-related changes to five types of physical activity: Active travel, Physical Education (PE), school breaktimes, active clubs (i.e. sports clubs, performing arts clubs, and outdoor activity clubs), and physical activity at home. Topic guides can be seen in the supplementary materials.

### Qualitative data analysis

The framework method was used to support the analysis of data [22]. This method was selected as it helped to reduce the large qualitative data set using summarised framework matrices that allowed information between participants to be compared easily. In addition, the summarised framework matrices served as tool whereby the multidisciplinary Active-6 team, each with different research experience, were able to actively participate and engage with the data. The data analysis process consisted of seven stages: 1) *verbatim* transcription by a university approved transcription service; 2) data familiarisation; 3) coding; 4) developing a working analytical framework using inductive and deductive codes; 5) applying the analytical framework; 6) charting data into the framework matrix; and 7) interpreting the data. In stage three, three researchers independently coded two transcripts for each participant group (parent and school contact interviews: RW, BT, and TR; child focus groups: RW, DH, and KS) using a mixture of inductive and deductive codes. Deductive codes were based upon key time points and activity types, allowing information related to these codes to be easily displayed in the framework matrices. The researchers then met to discuss interview content and interpretations and developed a separate code book for parents, school staff, and children that RW then applied to the remaining transcripts. Independent coding of transcripts was performed to support a deeper and more nuanced interpretation of the data.

A critical realist ontology was adopted in relation to the data [23]. Critical realism postulates that a reality exists independent of human beings, but recognises that human practices, such as culture and language, mediate and shape our experiences and understanding of reality. This was pertinent in this study which drew upon quantitative data and multiple perspectives of phenomena in order to provide a rich qualitative analysis.

## Results

**Fig. 2:**
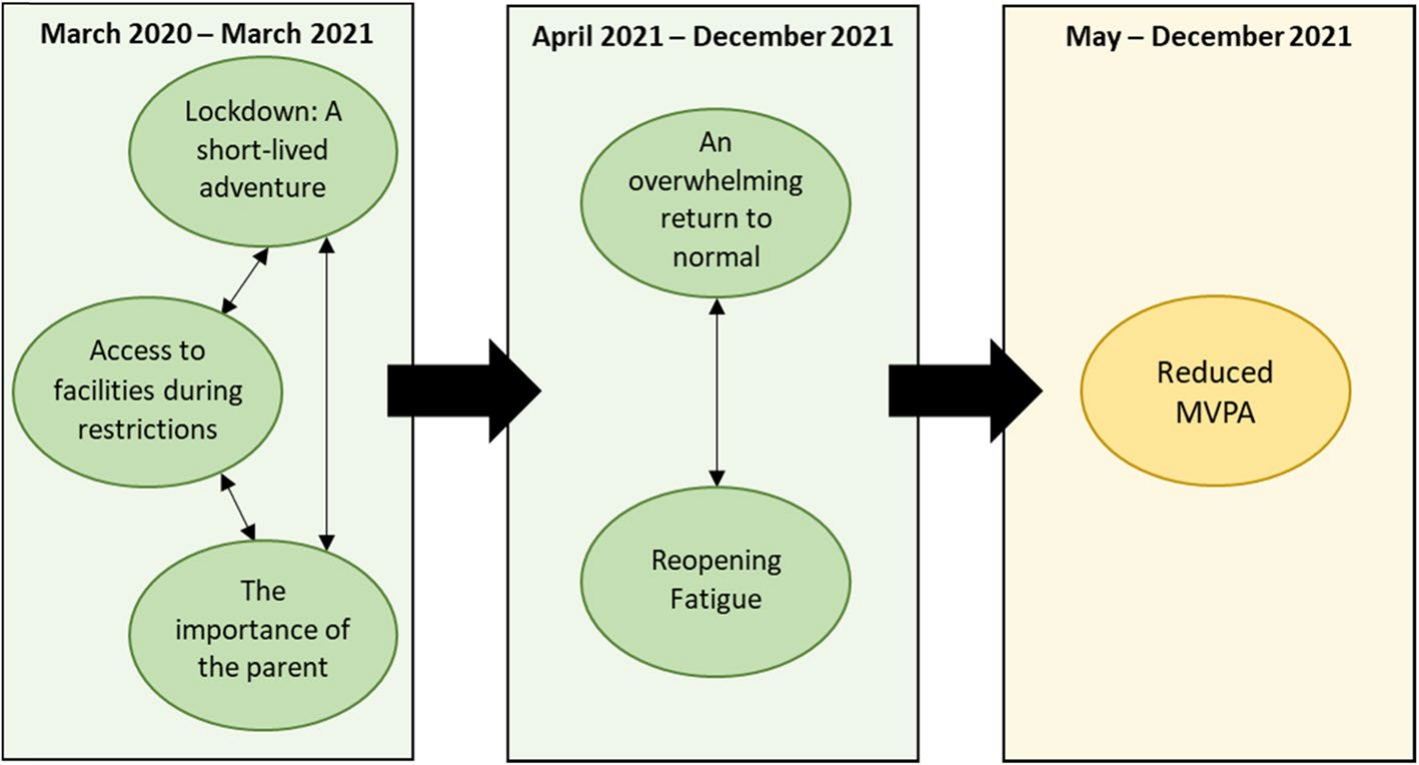
Thematic map with suggested relationships between themes and quantitative results. Note. Qualitative results are displayed in green; the reduction in MVPA reported by Salway and colleagues [14] is displayed in yellow

Five primary themes were generated that related to children’s physical activity behaviour over the course of the COVID-19 pandemic between March 2020 and December 2021. The first three themes relate to the March 2020 – April 2021 period and latter two to April 2021 – December 2021. The five themes were: 1) Lockdown: A short-lived adventure; 2) Access to facilities during restrictions; 3) The importance of the parent; 4) An overwhelming return to normal; and 5) Reopening fatigue. Theme definitions were constructed as short abstracts which illustrate the scope and boundaries of a theme’s multi-faceted underlying concept (Table 2). All five themes were reflected within data across the three participant groups. A thematic map with timepoints and hypothesised theme relationships is illustrated in Fig. 2.

**Table 2:** Theme names and definitions

	Theme name	Definition
1	Lockdown: A short-lived adventure	This theme explores the experience of a short-lived lockdown novelty where responsibilities and priorities shifted, both among families and within schools, leading to an increased motivation for wellbeing and physical activity. However, with prolonged restrictions and return to lockdown in winter 2020–21, these feelings turned to frustration and tedium. Responsibilities and priorities returned, in the form of academic and vocational pressures. This created a very challenging and inactive period for children and their families
2	Access to facilities during restrictions	The importance of access to appropriate physical activity facilities in the local and home environment was expressed in the data. Parents and children living in more rural communities, which was associated with greater levels of economic affluence, tended to be able to access more green space and had more space to utilise for physical activity within the home. Limited access to equipment at home, reduced the extent children enjoyed physical activity, particularly during the later stages of the lockdown, leading to disengagement from physical activity
3	The importance of the parent	This theme reflects the importance of the role of the parent in their child’s physical activity. The lockdowns and restrictions impacted children’s primary motivations for physical activity, such as having fun with friends, resulting in an increased need for external encouragement. In addition, the school’s inability to monitor physical activity at home resulted in an increased parental responsibility for their child’s activity
4	An overwhelming return to normal	A sense of an emotionally overwhelming transition between life in lockdown and other restrictions to an environment more closely representing a pre-pandemic normality, particularly during the return to school in September 2021, was expressed throughout the data. A significant increase in other children, apprehension of the unknown, and COVID-19 worries contributed to the feeling of being overwhelmed, which manifested itself as social conflict/withdrawal and some avoidance of active clubs
5	Reopening fatigue	An increased sense of physical fatigue and tiredness among children followed the second reopening in April 2021. Transitioning from a sedentary and secluded life in lockdown to a physically and emotionally challenging lifestyle contributed to these feelings of fatigue that impacted on motivation to participate in physical activities

### Theme 1: lockdown: a short-lived adventure

The first theme describes participants’ experiences of and response to the lockdowns and multiple changes to levels of pandemic-related restrictions and their impact on physical activity. Despite the strict lockdown enforced in March 2020 [19], participants evoked a strong sense of novelty and adventure at the sudden, and what was initially thought to be short-term, drastic changes to their lives. Parents conveyed a sense of disconnection from reality where previous responsibilities and priorities disappeared and spoke of feelings of excitement and freedom that energised and motivated families to focus on their wellbeing and physical activity.

*“I think it had been exciting…when we first went into lockdown in March 2020… at that point, we first thought it was only going to be for a short space of time… For us, it was a bit like going back to our childhood where you would go out and you would play out until late and not really care about what the time was…It was a very, very simple lifestyle… and there were no other competing priorities. It was just about, you know, whatever the activity was going to be… it became exciting as well” (Parent 15, female, IMD decile 10)*

Not only was this sense of novelty and disconnection from reality experienced at the family level, but also at the school level. In the initial stages of the pandemic, school contacts described a shift in the school curriculum from the usual academic pressures to activities focused on wellbeing, including physical activity. This led to a more flexible and relaxed home schooling for those not in the classroom, that was less time-consuming in the initial stages of the pandemic, adding to the sense of freedom and novelty that families were experiencing.

*“I think because it was all… I don’t want to use the word ‘fun’ but it was all new… I think it was a big thing at the beginning, it did tire off in the first lockdown but it was all new…” (School contact 6, School ID 71)*

*“We spent our mornings doing their schoolwork that they’d been set. We’d generally finished it by about lunchtime. The school isn’t breathing down our necks and saying, ‘Why haven’t they done all the spellings we asked them to do?’.” (Parent 18, female, IMD decile 4)*

Whilst participants described the initial lockdown as novel and exciting, the second, winter, lockdown was a more challenging period for families. In stark contrast to the response of the first lockdown, participants evoked strong feelings of stress, tedium, and boredom. Coinciding with the darker and shorter days, parents spoke of a gradual worsening of moods and motivation within the household. Many parents who were previously furloughed had now returned to their jobs, with many now working from home. School contacts described a transition in curriculum priorities that returned to core subjects, such as Maths and English. Consequently, home schooling became much more structured and reminiscent of a normal school day where children would be taught live lessons through video conference calling. Parents expressed that this created additional pressure and disempowered families to be active through a loss of autonomy caused by a more structured school day. School contacts also spoke of a need to catch up on missed learning due to pressures from government authorities to ensure that children were meeting the expected standard and prepared for standard attainment tests (SATS), a key milestone for Year 6 children in the UK.

*“I think that pressure to catch up and make sure the reading and the writing and the phonics was there… that became a priority again. It had always been a priority, but it became at the forefront…Catching up things that had been missed… the expectations were back from the government, as in like, ‘You will be taking assessments soon,’.” (School contact 3, School ID 64)*

The return of responsibilities of work and school required families to carefully balance a range of priorities, and a sense of just trying to survive and keep the family functioning through this challenging period was prominent in the data. Children expressed a longing for their pre-pandemic lives where they could spend time with friends and do more exciting activities. Families had exhausted their local areas and homes for new and engaging activities. Not only did this impact families’ motivation to be active, but also motivation in other aspects of their life, such as in work and school. Subsequently, the vast majority of parents described the winter lockdown as the least active period of the pandemic for themselves and their children.

*“…we did that [online exercise video] every day pretty much… until May [2020] half-term, and then one day, we all looked at each other and just thought, ‘I'm sick of this.’…In January [2021]* *, I think that was the point at which I just couldn't be bothered, and I think that probably rubbed off a little bit of the family. I lost my motivation at that point, and it was quite hard, I think, to keep going…Honestly, I had days when I just couldn't be bothered to get out of the house and move… I was getting through the day but only with the bare minimum...” (Parent 8, female, IMD decile 9)*

*“At the start of COVID, I was actually really excited because I got more time to do what I wanted to do outside school but nearer when we got into it and everyone realised that it wasn’t just going to be three weeks, it kind of got really boring...” (Child focus group 4, female)*

### Theme 2: access to facilities during restrictions

This theme illustrates the importance of access to appropriate physical activity facilities in the local and home environment. Participants suggested that the physical activity of children who were living in populous urban areas seemed to be disproportionately affected by the COVID-19 pandemic compared to those who lived in rural areas. Children and parents living in rural areas spoke of being able to utilise their local green space for physical activity, such as long family walks, with peace of mind that they could maintain COVID-19 health precautions. A strong sense of fortune and luck was expressed by those living in rural areas, with sympathy for families who lived in busy urban areas, which participants tended to link with lower levels of economic affluence. Participants suggested that living in more affluent rural communities during periods of COVID-19 restrictions generally allowed greater access to community recreation and play areas situated in their local area.

*“[physical activity] didn’t really change much because I have loads and loads of grass areas because I own horses and they all have their paddocks and everything. I could just run around basically where I wanted and I could ride my horses. I have dogs to go and walk with, I have [other pets] to play with. I didn’t really find it hard to be active in lockdown” (Child focus group 6, female)*

*“Luckily, we’re in a very rural location so we could do a big walk for an hour… every day… we have access to large open areas that we could use during those lockdown phases… we’re very fortunate to be in a rural location and a relatively prosperous location… Community-wise, there are plenty of play areas for them and recreation grounds.” (Parent 11, female, IMD decile 9)*

Participants also noted the impact of available space within the home on physical activity, which became awkward when managing limited space with other family members and pets. Participants also expressed the benefit of having an adequately sized garden where they could be active during lockdowns. One child who lived in an apartment described the problems with noise caused by doing physical activity in their living room and its disturbance to neighbours. Theme two was also reflected in access to equipment at home where children spoke of the variety of equipment they had access to at school, such as basketball hoops, balls, tennis rackets and courts, and how it increased the extent they enjoyed physical activity. Without access to such facilities and equipment at home, children quickly became bored, particularly during the later stages of the lockdown (Theme 1) and expressed significant difficulties at not having adequate facilities at home, leading to frustration and disengagement from physical activity.

*“Like how am I meant to do PE when it’s on a video? Cartwheeling all around the room while I’ve got a big fat dog on the floor, a couch in the way and other stuff like that. How am I supposed to do it?” (Child focus group 3, male)*

*“It’s difficult… [we] don’t have masses of room. We don’t have a garden… It’s all a bit awkward, trying to do it in the house with two people and cats running around and a sofa in the way… that certainly has an impact... Even when we do ‘Just Dance’ one of us has to stand where we’re going to hit the lampshade… space is an issue.” (Parent 21, female, IMD decile 6)*

### Theme 3: the importance of the parent

This theme focusses on the role of the parent and describes the importance of parental encouragement and support in their child’s physical activity over the various stages of the pandemic. Having fun with other children and its central importance in physical activity motivation among children was expressed throughout the data. However, as the lockdowns and restrictions were enforced, children were unable to play with other children, and their motivation to be active was greatly reduced. This was reflected in a lack of enthusiasm for virtual active clubs in comparison to physical attendance, even among those who were previously very engaged and motivated for physically attending their active club. Consequently, parents spoke of an increased need to act as an external motivator and encourage their children to do physical activity who would otherwise become very sedentary during the lockdowns. The role of the parent was highlighted by both parents and school contacts as one of the primary determining factors of a child’s activity levels during lockdowns and school closures, stemming from the parent’s value for physical activity. A reflective nature of the parent’s mood and motivation on their child was suggested in the data, something that many parents described decreased during the winter 2020–21 lockdown (Theme 1).

*“… [In] the first lockdown, the child’s physical activity was probably linked in with how much his or her parents saw the value of it. So, while the schools would put things on, how much was happening was really to do with those leading the families, the parents, really, and how much they were willing to push things and get the child to do things.” (School contact 8, School ID 24)*

*“If it wasn’t for me, I think it would have been really easy for them to slide into not doing it, not doing anything, really…” (Parent 14, female, IMD decile 2)*

This theme was also reflected by participants who spoke of challenges in relation to the availability of parents to encourage their child’s physical activity. This was salient among families with parents in full-time employment where some described an increased workload during the pandemic. Having a parent available to encourage physical activity among their children was expressed as a privilege that was linked to families living in more affluent sociodemographic areas who also had access to facilities that supported physical activity (Theme 2).

*“The demographic of kids here…are really privileged, and I think they come from families that are very keen to be healthy, and to stay fit….” (School contact 9, School ID 41)*

*“I think a massive factor for our family was the fact that I was very, very free for the children... So, when I compare with families where the parents were really trying to do a full day’s work, plus all the rest, that freedom that I had meant that my kids stayed much more active than most … But I think that was quite uncharacteristic in what I could see in neighbours and friends around us… I think that that was a privilege.” (Parent 1, female, IMD decile 9)*

The importance of the parent in encouraging physical activity during lockdown was further emphasised from the perspective of the school. Teachers felt they had very little control over what children would do at home during school closures. Although teachers were able to monitor more academic subjects, such as Maths and English, which had tangible outcomes, this was not possible with physical activity and PE. Consequently, what would have previously been the school’s responsibility to encourage PE was transferred to the parent.

*“I have no idea what was going on at home for that if I’m honest… That was probably the most fundamental difference [to when they were at school], that we could actually still keep an eye on them…” (School contact 5, School ID 25)*

### Theme 4: an overwhelming return to normal

This theme highlights the experience of an emotionally overwhelming transition from life in lockdown and other restrictions to the return to a life more closely representing pre-pandemic normality. Parents and children expressed feelings of fear and worry related to becoming infected with, or spreading, COVID-19. For many, their home became their sanctuary during a global disaster. A new normal was created within the limited boundaries and perceived safety of COVID-19 health measures.

Parents and children spoke of significant challenges when families needed to re-engage with a changed outside world, which was characterised by apprehension, fears and worries. As a result, both parents and children noted lasting changes to how they interacted with the outside world, such as wanting to avoid other people and lack of interest in finding new places and activities.

*“…sometimes you just feel like you don’t want to risk it going… because we don’t have a bubble anymore but you want to stay with your class. I know we can’t because other children are playing but you just want to try and stay so you feel a bit safer.” (Child focus group 1, male)*

*“[I feel like] a little bit of a shift or a change happened… about how you feel about going out and being around people in a way that was probably… a little bit off-putting… I still feel a bit like I want to hide in my house” (Parent 21, female, IMD decile 6)*

For children, the return to school after the summer holidays in September 2021 was a particularly difficult transition. During earlier phases of the pandemic, school contacts described the use of bubbles or pod systems that restricted children to interactions with their classmates only, as well as other COVID-19 health measures, such as reduced and staggered breaktimes, restrictions on access to equipment, and suspension of team sports and games. Many school contacts spoke of a desire and focus to return to normal among members of school staff, perhaps indicative of the tedium that characterised the winter lockdown (Theme 1), which eventually occurred in many schools following the 6-week summer break in September 2021.

*“We did get back to normal as much as we could, I think we were quite good at that…we felt like it was better to just get back to it. Apparently, that’s what our head had read… So we tried to make it as normal as possible where we could.” (School contact 6, School ID 71)*

Children described a strong sense of being overwhelmed by this transition and return to school. A combination of worries related to catching and spreading COVID-19 and an increase in the number of children in their social sphere created significant emotional and social challenges. Conversely, parents and school contacts described a resilience among children in relation to the negative impact of the pandemic.

*“I think kids are quite resilient and once they’re back out, they’re back out and they’re encouraged” (Parent 9, female, IMD decile 4)*

However, this was not reflected within the child focus groups who discussed significant emotional challenges, particularly during the winter lockdown (Theme 1). Social conflict with other children seemed to be prominent and a manifestation of feeling overwhelmed.

*“It felt really different because all I could see was people- there were some people in my class who I didn’t really like and were mean to me and I just didn’t like them and I didn’t want to go anywhere near them.” (Child focus group 4, male)*

Children described their return to active clubs as an overwhelming experience that characterised this transition. Parents and school contacts spoke of a staggered return of active clubs, with outdoor clubs able to restart sooner than indoor clubs. However, some school contacts described a delay to the resumption of afterschool clubs until the start of the academic school year in September 2021 due to an uncertainty related to the spread of COVID-19 and further lockdowns. Although many children expressed feelings of excitement at returning to active clubs, an apprehension of the unknown, such as who and what to expect in a post-COVID-19 society, was apparent. Worries related to having lost capability or ability following a return to active clubs were also common among participants. Some children spoke of emotional overwhelm and its effect on feelings of fatigue (Theme 5) and reduced motivation to participate in active clubs.

*“When restrictions went… I did go back to some of my clubs but then over a while I got really overwhelmed with all of them. Because we’d been at home for so long and I hadn’t been doing any clubs, it was a bit weird, that just all of a sudden that I was back doing swimming, gymnastics and all of those clubs so… [I] just said to my mum, ‘I’m getting too tired and I can’t really do it anymore.’.” (Child focus group 4, female)*

### Theme 5: reopening fatigue

The fifth theme describes an increased sense of physical fatigue and tiredness following the second reopening in April 2021. Participants spoke of a decrease in physical activity over the course of the pandemic, particularly during the winter lockdown (Theme 1). Both parents and school contacts noticed visible changes to body weight and a loss of fitness among some children; perceived to be an outcome of their sedentary time in lockdown. Parents evoked feelings of guilt if their child had gained weight, suggesting a belief that the responsibility of their child’s activity lies with the parent (Theme 3).

*“[Child name] in particular has gotten quite heavy since the last lockdown, and it really worries me… the amount of weight that he's put on. If I'm honest, I feel really quite guilty...” (Parent 8, female, IMD decile 9)*

*“When children came back to school we feel that a lot of them came back more unfit than before. I would say we saw that more in a visual way with some children… just looking at them around school and lunchtime… ‘Do they look unfit? Have they put on weight?’.” (School contact 2, School ID 72)*

As a result of a more sedentary life in lockdown, many children felt physically fatigued by the return to a busier, and closer to pre-pandemic, lifestyle. Transitioning from a strict winter lockdown to the return to school in April 2021 seemed particularly physically challenging (Theme 4). One member of school staff described some children who had lost fitness and gained weight during school closures had begun to avoid physical activity at school.

*“[some children] were trying to get out of [the] mile a day, suddenly started feigning a sore ankle or a sore foot or leg… you know that they were the ones that had put weight on over lockdown and had lost some of their fitness, so they were trying to get out of it” (School contact 5, School ID 25)*

Children discussed a strong sense of reluctance at the difficulty and effort of participating in physical activity post-lockdown. Where previously physical activities had been enjoyable for children, parents spoke of a need for them to persevere and overcome their discomfort, physical inability, and lack of enjoyment. Without such parent encouragement, the increased sense of fatigue and effort would likely lead to avoidance of these activities, again emphasising the role of the parent (Theme 3). Unstructured active play was suggested as a prominent type of physical activity that had been impacted by physical fatigue. Children described a reduced number of peers playing outdoors due to increased feelings of tiredness following the return to physical school attendance. As a result, more sedentary behaviours, such as electronic device use, became a more appealing activity. Despite lockdown ending in April 2021, a sense of enduring physical fatigue and tiredness was discussed in the focus groups (December 2021), perhaps due to the increased emotional challenges associated with the return to school and increased participation in active clubs in September 2021 (Theme 4).

*“[I think less people are playing outside] because they’re probably tired after school, and their legs probably just died…” (Child focus group 3, male)*

*“Swimming, I always used to enjoy it but now I don’t enjoy it as much because I find it really tiring and every week I say to my mum, ‘Can I not go?’ because I didn’t want to go because I just get tired and everything….I used to be really active but now, I don’t do much” (Child focus group 4, female)*

## Discussion

Findings from this study provide a novel multi-perspective qualitative insight into the effects of the pandemic on children’s physical activity behaviour in the UK. Mechanisms behind the observed reductions in MVPA among children who participated in Active-6 were explored. The five themes developed provide unique insight into the experiences of children aged 10–11 years living in England over the course of the pandemic between March 2020 and December 2021 and their influence on physical activity behaviour.

This qualitative analysis adds to the wider international narrative related to factors influencing observed changes in child physical activity over the course of the COVID-19 pandemic [14–18]. Previous qualitative research has explored this topic during earlier stages of the pandemic (March 2020 – January 2021) [24–28]. Although conducted in diverse settings and contexts, international qualitative research reflected many themes described in the current study. Appropriate outdoor and indoor space, access to equipment, and parental support have been identified as facilitators of physical activity during the pandemic and related restrictions [24–27], a finding echoed in a number of quantitative studies [13]. It is important to note that parental influences [29, 30] and access to facilities [30, 31] have been shown to influence physical activity behaviour among children prior to the COVID-19 pandemic. However, findings within the current study are suggestive of an increased importance during times of severe COVID-19 restrictions. Lockdowns, school closures, and travel limitations confined children to their homes for large periods of the day, limiting the area in which children were able to access facilities and significantly increased time spent with, and responsibilities of the parent. The notion of lockdown novelty, described in Theme 1 above, was also evidenced within other qualitative studies that were conducted in Canada and the USA, such as enjoying the increased opportunity to spend time with family in earlier stages of the pandemic [25] and subsequent tedium towards late 2020 [27, 28]. This is perhaps indicative of a cross-cultural response to extensive and prolonged COVID-19 restrictions.

Fears, anxieties, and nervousness of becoming infected or spreading COVID-19, adjusting to rules and guidelines, and loss of capability when returning to active clubs were also described in qualitative research in earlier stages of the pandemic [26–28], resonating with central aspects of Themes 4 and 5 described above. A key contribution of our work is that feelings of fear and worry were maintained following the easing of restrictions and have continued to contribute towards feelings of emotional overwhelm and physical fatigue that impacted physical activity behaviour.

The importance of social influences in physical among children has been well documented in pre-pandemic research [30, 32, 33]. Loss of social connections among children during periods of lockdown and isolation and its impact on wellbeing and physical activity has emerged in recent qualitative research [27, 28]. Results from the current study reflect a loss of social connection and suggest that, in the UK, social disconnection among children continued until September 2021 due to ongoing restrictions, such as reduced active club provision and school-level restrictions. Due to the impact of extended periods of social disconnection, children experienced significant emotional overwhelm and fatigue when they were eventually able to reengage with a society that more closely represented a pre-pandemic normality. This led to avoidance of physical activity, particularly unstructured active play, which provides explanation for the observed decrease in MVPA and increase in sedentary time among children who participated in Active-6 [14]. The narrative relating to changes to active play within two recent qualitative studies were mixed. Pelletier and colleagues described a shift in physical activity behaviour towards unstructured active play with siblings and family [28]; whereas, Szpunar and colleagues reported a decrease in unstructured active play due to a loss of other children to play with [27]. However, these differences might be explained by cultural and contextual differences between the two Canadian populations studied, *i.e.* Szpunar and colleagues exploring a largely urban-dwelling population, whilst Pelletier and colleagues participants were situated in a more rural environment. Nevertheless, in the context of the current study, reductions in unstructured active play were prominent.

### Study implications

Table 3 highlights key findings and implications from this study. The primary message is the emotional and physical challenges experienced by children following the easing of COVID-19 restrictions are key contributors to the decreased MVPA and increased sedentary behaviour identified in a related quantitative study [14]. It is important to develop new approaches to provide support for children to address these issues. Moreover, findings stress the mental challenges associated with prolonged COVID-19 restrictions, leading to increased sedentary behaviour and motivation for physical activity. If further COVID-19 restrictions are considered in the future, it is vital that policy makers are aware of their impact on mental and physical health. The importance of the parent and access to appropriate physical activity facilities, such as access to green space and sports equipment, during periods of lockdown were prominent themes in the data. Thus, ensuring that families have the appropriate skills, knowledge, and facilities is vital for promoting physical activity among children during periods of lockdown and restrictions.

**Table 3:** Key findings and implications

Key finding	Implications
Post-COVID-19 challenges	Despite easing of restrictions, returning to normality presented many challenges for children that impacted their physical activity, predominantly emotional overwhelm and fatigue. It is important that practitioners consider these challenges and provide appropriate support for children
Reductions in unstructured active play	Data suggested that active play behaviour, such as children playing with friends outside, has been negatively impacted by the COVID-19 pandemic, contributing to a net decrease in MVPA among children. Strategies to promote active play are warranted
COVID-19 restriction tedium	In later stages of the pandemic, COVID-19 restrictions became mentally challenging for many families, leading to increased sedentary behaviour. Policy makers should be aware of the impact of restrictions on mental and physical health
The importance of the parent	Parental encouragement and support were important for child activity levels, especially during periods of lockdown. Providing parents with relevant skills and knowledge to enable them to facilitate enjoyable physical activities for their child during periods of COVID-19 restrictions is needed
Access to physical activity facilities	Ensuring that children and families have access to appropriate physical activity facilities is warranted, particularly during periods of restrictions. Ensuring that children have access to outdoor green space and providing families with equipment, such as sports equipment, will help avoid repetitive physical activity and alleviate boredom when COVID-19 restrictions reduce opportunities. Policy makers should be aware of the impact of reduced access to appropriate facilities on child physical activity behaviour

### Strengths and limitations

This study had two main strengths. Findings extended the collective international body of knowledge to include later stages of the pandemic (March 2020—December 2021), where previous research has focused on periods before January 2021. This allowed the inclusion of timepoints where COVID-19 restrictions were gradually eased, providing insight related to their impact following the reopening of society. In addition, exploring phenomena and themes through a multi-perspective lens provided a more nuanced and rich analysis. To date, qualitative research exploring this topic has predominantly focused on the perspectives of the parents [24, 26], parents and children [25, 27], and families (at least one parent and child) [28]. The design of the current study is unique in that it included the school environment/perspective, allowing a large part of children’s pandemic experience that parents may have limited knowledge of to be integrated into the analysis.

It is important to note this study’s limitations. The parent sample were entirely female and predominantly active, with only one parent categorised as having low MVPA levels. The experiences of less active and male parents are missing from the analysis. Despite concerted efforts to recruit a diverse range of parents, this was not possible in the current study. However, the child focus group participants were drawn from a range of contexts and backgrounds which provided a variety of perspectives. Nevertheless, future research would benefit from the inclusion of male parents and those with lower activity levels. It is also important to note that the sample recruited in Active-6 was predominantly white-British [14], as reflected in the demographics of participants of this study. Thus, further research exploring findings in more diverse populations is warranted.

## Conclusion

The COVID-19 pandemic has had a marked impact on children’s physical activity in the UK despite the removal of restrictions. This qualitative analysis explored the experiences of children throughout different stages of the pandemic, suggesting that the novelty and excitement felt during the initial stages of lockdown declined as varying levels of lockdowns and restrictions continued. This created an increasingly challenging period for parents and their children, whose lives became progressively demanding, isolated, and sedentary. During these periods of strict lockdowns and restrictions, the importance of parental encouragement and access to appropriate facilities in the local and home environment were facilitators of physical activity, with participants perceiving those living in less economically affluent and urban areas being disproportionately negatively impacted. Following the easing of COVID-19 restrictions, emotional overwhelm and physical fatigue among children, stemming from a sedentary and socially isolated life in lockdown and other restrictions, were key contributors to the decreased MVPA and increased sedentary behaviour that was observed in a related quantitative study. Future research to monitor long-term changes to physical activity behaviour in later stages of the pandemic and strategies to address reductions are vital in the wake of the COVID-19 pandemic.

## Supplementary Information

**Additional file 1: Supplementary File.** Interview and focus group topic guides.

## Data Availability

As the Active-6 project is still ongoing, data are not currently available. Data will be made available in the University of Bristol’s data repository at the end of the project (https://data.bris.ac.uk/data/).

## Abbreviation

MVPA: Moderate to vigorous physical activity
