# Supplementary material for: A multi-perspective qualitative exploration of the reasons for changes in the physical activity among 10–11-year-old children following the easing of the COVID-19 lockdown in the UK in 2021
Source: Int J Behav Nutr Phys Act. 2022 Sep 5;19:114. doi: 10.1186/s12966-022-01356-3 (PMC9444096; doi:10.1186/s12966-022-01356-3)
Supplement: Supplementary file 1 — Additional file 1: Supplementary File. Interview and focus group topic guides. [file 12966_2022_1356_MOESM1_ESM.docx]

**A multi-perspective qualitative exploration of the reasons for changes in the physical activity among 10–11-year-old children following the easing of the COVID-19 lockdown in the UK in 2021**

Robert Walker_,_ Danielle House_,_ Lydia Emm-Collison_,_ Ruth Salway, Byron Tibbitts_,_ Kate Sansum_,_ Tom Reid_,_ Katie Breheny_,_ Sarah Churchward_,_ Joanna G Williams, Frank de Vocht, William Hollingworth, Charlie Foster and Russell Jago PhD

**Supplementary File: Interview and focus group topic guides**

This file includes the guides for: A) Parent interviews; B) School Staff; and C) Student focus groups.

1. **Parent interview guide**

**Introduction and consent**

Thank you for agreeing to take part in this interview, your views and opinions are really important to us.

In the interview today, I would like to talk about three main points:

1. You and your child’s physical activity patterns and how these may have been impacted by COVID-19.
2. Factors that you feel you have caused any changes
3. Whether you think there is anything that can be done to help support you and your child’s physical activity

We are really interested in your honest opinions, we are not here to judge you, and we do not want you to feel like you should answer any of the questions in a certain way. There are no right or wrong answers, and as much detail you can give on the topics as possible is really appreciated.

Before we get started, I’d like to go over some important information regarding the interview and your data:

- I will be **recording the conversation** to help us remember what you said
- You can ask for the recording to be stopped at any time
- After we have written a report about all the opinions we have heard from the parents taking part, the recordings will be destroyed
- We will also change any names or identifying information so none of the information that is written down and recorded can be connected to you in any way
- Please remember that you can interrupt the interview at any point if you need to.
- If you do not want to answer a question please say so.
- Are you comfortable to proceed with the interview?

If the parent says yes the recording will start. As noted on the information sheet the interview will be recorded.

- For the recording, can you please confirm that you have been given information explaining about the study and that you understand what this project is about?
- Can you please confirm that you have had the opportunity to ask questions and discuss this study?
- Can you please confirm that you are aware that the interview data will be stored anonymously and securely for 20 years?
- Can you please confirm that you are aware that you are free to withdraw your data from the study at any time up to three weeks after this interview has taken place **[X DATE]**, and that you do not need to give a reason for withdrawing?
- And finally, can you please confirm that you are happy to take part in the interview?

**Icebreakers**

1. Discussion of physical activity definition (4 domains)
   1. Leisure-time
   2. Transport
   3. Household
   4. Occupational
2. Can you tell us what your child in Year 6’s favourite physical activity/physically active thing to do is and why do you think this is their favourite?
3. Can you describe your favourite physical activity/physically active thing to do is and why do you think this is their favourite?

**Changes in PA and device/screen time over COVID pandemic**

***Pre-lockdown***

1. How would you describe your activity levels before the first lockdown?
   - Did you often use active modes of transport?
   - Were you part of any active clubs or have any active hobbies?
   - Are you very active around the house?
   - If you are employed, is your work active?
2. How active was your child before the first lockdown?
   - Did your child use active modes of transport to school?
   - Did they participate in any active clubs or hobbies?
   - If you feel you can answer, were they active during playtimes?

***First school closure/lockdown 1 (March 2020)***

1. Did your child attend school at this time? (i.e. parents were key workers)
   1. If so, to what extent do you feel this influenced their activity levels?
2. To what extent do you feel the first lockdown/school closure influence your activity levels?
3. How about your child’s?
4. What were the key factors that influenced any changes to your/your child’s PA at this time?

Pool of prompts (exact prompts used will be guided by the contact’s role/previous discussions):

| **Policy** | |
| --- | --- |
| 1. To what extent do you feel that any policy/legal restrictions associated with COVID-19 effected your/your child’s (i.e. lockdowns, social distancing)? | □ |
| **Environment** | |
| 1. To what extent did you/your child’s opportunities to be physically active outside of school change at this time?    1. How did you feel about these changes? | □ |
| 1. How do you feel any changes to school or school curriculum PE influence your child’s activity levels?    1. How did you feel about these changes?    2. Did you feel supported in doing PE from home? | □ |
| **Strategies to promote PA** | |
| 1. To what extent did you feel supported and encouraged by the school or other organisations to be physically active at this time? | □ |
| **Attitudes** | |
| 1. Can describe your attitude and thoughts towards physical activity at this time? Had it changed? | □ |
| 1. To what extent do you feel that the school’s attitude towards PE and other physical activities changed at this time? | □ |
| 1. To what extent do you feel that being less or being more active was the norm during lockdowns, or did it stay the same? Was this the same for both child and parent? | □ |
| **Social** | |
| 1. To what extent do you feel your child’s active play with their friends changed at this time? | □ |
| 1. To what extent did social distancing and not being able to see other people influence your/your child’s physical activity? | □ |
| **Motivation** | |
| 1. To what extent do you feel that your/child’s motivation for physical activity changed at this time? | □ |
| 1. Have you noticed changes related to confidence in your ability to do PA in your child? If so, how did it change? | □ |
| Emotion | |
| 1. Thinking back to how you felt at this time, to what extent did your/your child’s feelings influence your activity levels? For example, was lockdown 1 a novelty that made you want to be more active or were you very worried and did not want to leave your house? | □ |

***First return to school (Autumn 2020)***

1. Did your child attend school this time? If so, what had changed?
2. To what extent did the lifting of the lockdown and return to school influence your physical activity levels?
3. How about your child’s?
4. What were the key factors that influenced any changes to your/your child’s activity at this time?

***Second school closure (January – March 2021)***

1. Did your child attend school at all at this time?
   1. If so, what had changed?
   2. To what extent do you feel this influenced their activity levels?
2. Thinking back to the first lockdown/school closure, to what extent did your/your child’s activity levels differ in the second lockdown/closure compared to the first?
3. Can you please describe any differences between this lockdown/school closure and the first?

***Second (most recent) reopening of schools (April – July 2021)***

1. To what extent did the most recent return to school influence your activity levels?
2. How about your child’s?
3. What were the key factors that influenced any changes to your/your child’s PA at this time?

***Other factors influencing PA***

1. Are there any other factors that we haven’t discussed that you think have influenced your/your child’s activity levels over the course of the pandemic so far?

***Electronic device use/screen time***

1. How has your child’s use of electronic devices changed along the way – total time and how they use them (i.e. for school work/socializing/gaming or watching things)?

**Current solution-focused ideas for themselves and their child**

1. Evidence from government reports and other research suggests that physical activity has decreased since the pandemic started. To what extent does this match up with your experiences?
2. Are there any barriers that are ***currently*** preventing you/your child from being more active?
   1. How and who could support you to remove these barriers?
3. Is there anything that could help to boost your/your child’s motivation to be active?
4. Is anything needed to help enable you/your child to do more of the active things you/they wish to do?
   1. Where/when/how should it be available?
   2. How could the school support it?
   3. How could local communities/local government support it?
   4. How could national government support?

**Closing statement**

- Is there anything else you’d like to tell us about the things we talked about today?
- Do you have any questions for me?
- We appreciate you sharing your thoughts and opinions with us!

1. **School staff interview guide**

**Introduction**

- Thank participant for taking part in the interview and introduce myself
- Describe the purpose of the ACTIVE-6 project and the interview
- Discuss definition of PA
- Key components of the interview
  - Discuss any changes within the school that may have influenced PA throughout different phases of the pandemic
  - Your thoughts on how these changes may have influenced levels of physical activity among Year 6 children
  - The approach of the school to promote physical activity during and after national school closures
  - Whether there are any key lessons or suggestions for the future
- Emphasise that there are no wrong answers and that I am not here to judge, but to explore their experiences related to what changed and how this may have impacted PA among Year 6 children.
- Confidentiality procedures
  - I’d like to tell you that I will be **recording the conversation** to help me remember what you said.
  - You can ask for the recording to be stopped at any time.
  - After the interview, the recording will be transcribed/written up word for word
  - After the interview has been transcribed, the audio recordings will be destroyed
  - In the transcripts, all identifying information will be removed, so nothing you say can be connected to you in any way
  - Anonymised transcripts will be held safely and securely for 20 years
  - Please remember that you can interrupt the interview at any point if you need to and that you are free to withdraw your data from this study up to three weeks after the interview. You do not need a reason for withdrawing.
  - You are also completely free to not answer any of the questions if you do not want to you
  - Do you have any questions for me?
  - Are you happy to proceed with the interview? **If yes, begin the recording**
- For the recording, can you please confirm that you have been given information explaining about the study and that you understand what this project is about?
- Can you please confirm that you have had the opportunity to ask questions and discuss this study?
- Can you please confirm that you are aware that the interview data (transcriptions) will be stored anonymously and securely for 20 years?
- Can you please confirm that you are aware that you are free to withdraw your data from the study at any time up to three weeks after this interview has taken place **[X DATE]**, and that you do not need to give a reason for withdrawing?
- And finally, can you please confirm that you are happy to take part in the interview?

**Icebreaker/Demographics questions**

The first few questions will help me get to know you a little better, what your role is in the school, and your thoughts about PA.

1. Please tell me what role you hold in the school and what level of influence you have over school policies surrounding physical activity.
2. Are you a qualified teacher? If so, how long have you been teaching?
3. Does your role involve teaching PE? (If so how often?)
4. What kind of involvement do you have with the extracurricular programme at your school?
5. What personal value do you place on physical activity?

**Changes in/factors influencing physical activity among year 6 pupils**

The next set of questions discuss any differences/changes in the school and how these may have influenced PA among Year 6 children over the course of the pandemic so far.

***Pre-COVID 19***

1. How active would you say Year 6 pupils were at school before the pandemic?
   1. How active were playtimes, generally?
   2. What was participation in active after-school clubs like? An active after-school club is a club at your school that is all about playing a sport or being active.
   3. How frequently did pupils use active modes of travel to get to and from the school? (*e.g.* walking, cycling, scooting)

***First school closure (mid March)***

1. Can you describe any differences/changes within the school that might have influenced physical activity among Year 6 pupils at this time?

Pool of prompts (exact prompts used will be guided by the contact’s role/previous discussions):

| **Policy** | |
| --- | --- |
| 1. Can you describe any PA-related school policy changes at this time?    1. What did you think about these policy changes?    2. To what extent did you feel supported by the school/government in implementing these policies?    3. Can you outline any funding changes/problems that influenced school PA at this time?    4. How did you feel about these changes? | □ |
| **Environment** | |
| 1. To what extent did opportunities for PA within the curriculum change?    1. What did you think about these changes? | □ |
| 1. To what extent did extracurricular opportunities for PA change?    1. What did you think about these changes? | □ |
| 1. To what extent do you feel that staff were supported in delivering PA at this time? | □ |
| **Strategies to promote PA** | |
| 1. Can you outline any strategies your school created to promote PA during school closures?    1. Can you describe the decision making/design process for these strategies? What did you think about them?    2. What were your experiences/thoughts of these strategies? How easy/difficult was it?    3. How effective do you think they were in achieving their aims?    4. To what extent would you change any of these strategies? | □ |
| **Attitudes** | |
| 1. To what extent do you feel that any attitudes towards PA within the school changed at this time? | □ |
| 1. To what extent do you feel that any attitudes of parents towards PA changed at this time? | □ |
| 1. Can you describe the school’s priorities during the first school closure and their relation to school physical activity? | □ |
| **Social** | |
| 1. To what extent do you feel active play among pupils changed at this time? | □ |
| **Motivation** | |
| 1. To what extent do you feel that motivation for PA changed within the school this time? (i.e. among pupils or school delivering PA) | □ |
| 1. To what extent do you feel confidence in ability to do PA changed among Year 6 pupils at this time? | □ |

1. If you feel you can, can you comment on the implication of these changes for overall physical activity levels among Year 6 pupils? (Frequency or type differences?)
2. How many children attended school in-person during the first school closure? (How many Year 6 – approx.)
3. To what extent do you think attending school in-person influenced their levels of school-related PA?

***First return to school (Autumn 2020)***

1. Can you describe any differences/changes within the school that may have influenced PA among Year 6 pupils following the first return to school?

| **Environment** | |
| --- | --- |
| 1. To what extent do you feel that any COVID restrictions in the school influences activity levels?    1. Bubbles    2. Restrictions on movement    3. Restriction on play equipment    4. Spaced out desks    5. Outdoor learning | □ |

1. If you feel you can, can you comment on the implication of these changes for overall physical activity levels among Year 6 pupils?

***Second school closure (January - March 2021)***

1. Thinking back to any differences/changes within the school that impacted PA during the first closure, to what extent did any changes during the second closure differ to the first?
2. Can you describe any differences between this school closure and the first?
3. To what extent do you feel that activity levels among Year 6 pupils differed during this school closure compared to the first?
4. Can you tell me about any differences in the number of children attending school in-person during the second school closure compared to the first?
5. To what extent do you feel attending school in-person influenced their levels of school-related PA?

***Second/most recent return to school (April 2021)***

1. Can you describe any changes within the school after the return to school in April that may have influenced physical activity among Year 6 pupils?
2. If you feel you can, can you comment on the impact of these changes might have had on overall physical activity levels among Year 6 pupils?
3. Looking back over the course of the pandemic, to what extent did your role differ and change in relation to physical activity?
   1. How about the role of other staff members?

***Other factors influencing PA among year 6 pupils***

1. Are there any other factors that we haven’t discussed that you think have influenced or changed levels of PA among Year 6 pupils throughout the course of the pandemic so far?

**Key lessons and recommendations related to encouraging PA among pupils during a pandemic**

The final set of questions explore your thoughts on any lessons learned and future recommendations.

1. Can you describe any key lessons the school learned related to encouraging PA among year 6 pupils from the experience gained during the course of the pandemic so far?
2. To what extent do you plan to continue using any changes/strategies related to PA made during the pandemic?
3. To what extent do you think anything could have been helpful when schools first closed for parents or staff to help promote PA?
4. If we were put in a similar situation again, is there anything you think you/your school would have done differently in terms of supporting pupil physical activity

**Closing statement**

- Is there anything else you’d like to tell us about what we have talked about today?
- Do you have any questions for me?
- Thank participant and end interview.

1. **Student focus group guide**

**Introduction (10-15 minutes)**

- Thank children for participating in the ACTIVE-6 project
- Introduce myself
- Explain why this project is important and what it means for them

***Active travel (10 minutes)***

1. Who here has ever walked, biked, or scooted to school before?
2. Who has changed how they travel to school since coming back after lockdown?
3. If you could choose anyway to travel to school, what would you choose? Why? What is stops you from doing that?

**Physical activity during school time (15 minutes)**

***School PE***

1. What is everyone’s favourite thing to do in PE? What did you like about it?
2. How did everyone feel about PE before, during, and after?
3. Has your feelings changed toward PE since after the lockdowns?

***Breaktime***

1. Who likes playing in the playground? What kind of things do you do?
2. Did anyone miss not being able to play in the playground when schools were closed?
3. How was it going back into the playground after lockdown?
4. Did it feel different?

**Physical activity outside of/after school (15 minutes)**

***Activity***: Ask children to draw themselves doing the physical activity they do the most at home. For example, playing with friends and going on walks with family. Ask them to add words around it to describe how it makes them feel. Children will have 2 minutes to draw their best stick figure-style drawing.

***Active clubs***

1. Who did an active club, such as football or swimming, before coronavirus? **(SHORT)**
2. After schools were closed, what happened to your active club? Did it continue online or was it cancelled?
3. What was it like going back to active clubs after lockdown?
4. Did anybody choose not to go back to their active club because of COVID?
5. Who has started an active club after coming back to school? What made you want to start one?

***At home/screen time***

1. How did everyone spend their free time during lockdown?
2. Was this different to what you used to do before lockdown?
3. Are you still spending your free time that way?
4. Does anyone think their parents rules about screen time changed?

**Closing statement and questions**

- Thank all the children and tell them they have been a big help with our research
- Overall, is there anything anyone would like to talk about or mention that we haven’t already discussed?
- Are there any questions?
- Say goodbye and end focus group
